# Perinatal Outcomes of Singleton, Twin and Triplet Gestations after Oocyte Donation: A Retrospective, Population-Based Cohort Analysis

**DOI:** 10.3390/children11080962

**Published:** 2024-08-10

**Authors:** Or Eliner, Roni Rahav Koren, Hila Shalev Ram, Mattan Levi, Einat Haikin Herzberger, Amir Wiser, Netanella Miller

**Affiliations:** 1 IVF Unit-In Vitro Fertilization Unit, Department of Obstetrics and Gynecology, Meir Medical Center, Kfar Saba 4428164, Israelamir.wiser@clalit.org.il (A.W.);; 2 School of Medicine, Faculty of Medical and Health Sciences, Tel Aviv University, Tel Aviv 6997801, Israel

**Keywords:** oocyte donation, small for gestational age, preterm birth, pregnancy-induced hypertension

## Abstract

Background/Objectives: Although high live birth rates are associated with oocyte donation (OD), these pregnancies are associated with increased obstetric and perinatal risks. This study evaluated maternal and neonatal risks after OD compared to in vitro fertilization (IVF) with autologous oocytes, and to spontaneous pregnancies (SPs), among singletons, twins and triplets. Methods: A retrospective, large, population-based cohort study was conducted based on electronic data from Maccabi Healthcare Services. A total of 469,134 pregnancies were grouped according to the mode of conception. The main outcome measures were preterm birth (PTB), small for gestational age (SGA) and pregnancy-induced hypertension (PIH). The data were analyzed separately for singletons, twins and triplets. Results: The mean maternal age was older in the OD group compared with the IVF and SP groups (singletons: 39.7 ± 4.1 vs. 34.5 ± 4.8 and 31.7 ± 5.3 years; twins: 39 ± 4.6 vs. 32.6 ± 4.4 and 31.2 ± 5.1 years; and triplets: 35.6 ± 2.5 vs. 32 ± 3.9 and 29.7 ± 5 years). The mean gestational age was younger among the OD group compared to the SP group (singletons: 37.5 ± 3 vs. 39 ± 2 *p* = 0.001, and twins: 35 ± 3 vs. 36 ± 2.5 *p* = 0.001). Higher rates of PTB < 37, PTB < 34 and PTB < 28 weeks were found among OD singletons. Multivariable logistic regressions for PTB < 37 weeks and SGA in singletons demonstrated that OD and IVF are significant risk factors (OR = 4.1, 95%CI = 3.3–5.2; OR = 4.3, 95%CI = 4.1–4.6; OR = 1.9, 95%CI = 1.3–2.6; OR = 2.2, 95%CI = 2–2.4, respectively). Significantly higher rates of PIH were demonstrated among the OD vs. IVF and SP groups in singleton (4.3% vs. 1.7% and 0.7%) and in twin pregnancies (7.5% vs. 4.3% and 3.4%). Conclusions: OD pregnancies are at increased risk for PTB, SGA and PIH.

## 1. Introduction

Oocyte donation (OD) has become an integral part of assisted reproductive technologies (ART) and has been used for more than three decades [1,2]. Recipients of OD are usually of advanced age and have undergone unsuccessful IVF treatments using autologous oocytes [3,4]. 

Although the rates of success in terms of pregnancy and live birth rates are high [4,5], OD pregnancies are at higher risk for maternal and obstetric morbidity [5,6,7,8,9,10,11,12].

Studies have demonstrated that OD pregnancies have an independent, increased rate of pregnancy-induced hypertension (PIH) compared to autologous oocyte IVF cycles and spontaneously conceived pregnancies (SPs) [5,6,7,8,9,12,13]. A systematic review and meta-analysis including more than 16,000 OD pregnancies demonstrated higher risk for pre-eclampsia (PE) for both singletons and twins, with adjusted ORs (aORs) of 2.11 and 3.31, respectively [9]. Another study assessing pregnancy and perinatal risks among OD vs. autologous IVF and non-IVF conceptions among twins also demonstrated an increased risk of PE among OD pregnancies [12]. Furthermore, an increased rate of Cesarean delivery was demonstrated compared to autologous oocyte IVF pregnancies [5,14,15]. This increased morbidity may be related to several factors, including advanced maternal age, multiple pregnancies and specific diseases related to infertility [10,16].

Despite the apparent increase in hypertensive disorders and Cesarean delivery rates, data regarding other perinatal outcomes of OD pregnancies, especially preterm birth (PTB) and low birth weight (LBW), are inconsistent [6,7,9,10,11,12,17,18,19]. Gibbons et al., Malchau et al. and Schwartz et al. reported poor perinatal outcomes, including higher rates of preterm birth and low birth weights (LBW) [7,18,19]. Also, Storgaard et al. in their systematic review and meta-analysis showed higher rates of PTB among OD pregnancies vs. autologous IVF in singletons [9]. On the other hand, Klatsky et al. reported similar rates of preterm birth and LBW between OD and autologous oocyte pregnancies in patients aged 35 and older [6]. Other studies also failed to demonstrate a difference in the risk for PTB between OD and autologous IVF and/or spontaneous conceptions [12,17].

Because of the inconsistency regarding some of the pregnancy and perinatal complications associated with OD, among singletons and multiple gestations, the objective of this study was to describe the obstetrical and perinatal outcomes of children conceived after OD compared to IVF with autologous oocytes and to SPs, including separate analyses of singleton, twin and triplet pregnancies.

## 2. Materials and Methods

### 2.1. Study Design

This retrospective, large, population-based study compared the obstetrical and perinatal outcomes of neonates born after OD, IVF, and SP.

The study was based on electronic data from Maccabi Healthcare Services, a 2.5-million-patient integrated care organization in Israel, which covers 25% of the population in the country [20]. Data were collected from 2000 through 2019 and were fully anonymized before analysis. All diagnoses were defined according to the International Classification of Diseases, 9th Revision, Clinical Modification (ICD-9-CM), as assigned by each patient’s obstetrician.

### 2.2. Study Population

In order to address pregnancy and perinatal complications according to the mode of conception, patients were grouped into three groups according to the mode of conception: OD, IVF or SP. For each group, the data were analyzed separately and compared for singletons, twins and triplets. Patients’ demographic parameters, including their socioeconomic status (SES) (determined according to the poverty index of the patient’s address enumeration area as defined by the 2008 Israeli national census statistics), BMI and number of previous deliveries, were collected. Perinatal outcomes, including birth weight; birth week; the child’s sex; preterm birth (PTB) < 37; PTB < 34 weeks; PTB < 32 weeks; and small for gestational age (SGA), calculated as the 10th percentile of Dolberg’s birthweight curves for singletons and twins [21] (a curve for triplets is not available), were analyzed. Obstetrical outcomes, including hypertensive disorders in pregnancy (pregnancy-induced hypertension (PIH), which includes chronic hypertension (CHTN), pre-eclampsia and gestational hypertension (GHTN)); pre-gestational and gestational diabetes mellitus (DM and GDM); and malpresentation, were also evaluated.

Patients included in the study were patients aged 18–45 years old who had sufficient data regarding their obstetrics and perinatal complications and were treated with a single mode of conception (OD, IVF or SP).

Patients were excluded if they were under 18 years or above 45 years old and/or were treated with more than one mode of conception. Neonatal records lacking well-documented data regarding gestational age (GA) and birth weight were also excluded.

Furthermore, miscarriages and deliveries before 24 weeks were excluded.

### 2.3. Outcome Measures

The primary outcomes were SGA (10th percentile of Dolberg’s birthweight curves for singletons and twins [21]) and PTB < 37 weeks. The secondary outcomes included diagnoses associated with high-risk pregnancies, according to ICD-9-CM diagnoses assigned by the patients’ obstetricians, and included PIH, DM and GDM.

### 2.4. Statistical Analysis

Data were analyzed using the SPSS version 24.0 package for Windows (SPSS, Inc., Armonk, NY, USA) and Phyton 3.7.9. Categorical variables are presented as numbers and rates (percentage), and continuous variables are presented as means and standard deviation. *p*-values were calculated using a t test or a χ^2^ test for continuous and categorical variables, respectively.

To estimate the risks of PTB < 37 weeks and SGA, multivariable logistic regression analyses were performed. The analyses were adjusted for maternal age, BMI, SES, OD, IVF, PIH (including CHTN, pre-eclampsia and GHTN), and DM and GDM. *p*-values < 0.05 were considered significant.

### 2.5. Ethical Considerations

This study was conducted in accordance with the Declaration of Helsinki and approved by the Institutional Review Board of the Maccabi Health Maintenance Organization, number 0046-18, on 1 May 2018.

## 3. Results

### 3.1. Maternal Characteristics

The study population consisted of 483 pregnancies conceived with OD, 12,579 pregnancies conceived after IVF with autologous oocytes, and 456,072 SPs.

The maternal background characteristics are described in Table 1.

The mean maternal age was significantly older among the OD group compared with the IVF and SP groups (singletons: 39.7 ± 4.1 vs. 34.5 ± 4.8 and 31.7 ± 5.3 years; twins: 39 ± 4.6 vs. 32.6 ± 4.4 and 31.2 ± 5.1 years; and triplets (35.6 ± 2.5 vs. 32 ± 3.9 and 29.7 ± 5 years). The mean maternal age of patients who had undergone IVF was significantly higher than that of patients who had undergone SP for singletons, twins and triplets (Table 1). Patients in the OD group had a higher SES compared to those in the IVF and SP groups for singletons (7 ± 1.5 vs. 6.7 ± 2 and 5.9 ± 2 years), twins (7.2 ± 1.6 vs. 6.6 ± 1.8 and 6.4 ± 2) and triplets (7.2 ± 0.4 vs. 5.8 ± 1.8 and 5 ± 1.8 years). The OD group had a higher rate of nulliparity among singleton pregnancies (65%) compared to the IVF (0.6%) and SP (51.8%) groups. BMI was similar among the OD, IVF and SP groups, for single and multiple gestations (Table 1).

### 3.2. Perinatal Outcomes

The perinatal outcomes of children born after OD, IVF and SP are shown in Table 2.

The mean GA was significantly lower among OD pregnancies compared to SPs for both singleton (37.5 ± 3 vs. 39 ± 2 weeks, *p* = 0.001) and twin (35 ± 3 vs. 36 ± 2.5 weeks, *p* = 0.001) gestations. Triplet OD pregnancies had a higher mean GA compared to IVF and were comparable to SPs (Table 2). Comparing the rates of PTB < 37 weeks, PTB < 34 weeks and PTB < 32 weeks between OD and SP for singletons and twins, higher rates were demonstrated among the OD groups (for singletons, GA < 37: 24% vs. 5.6%, *p* = 0.001; GA < 34: 7.5% vs. 1.1%, *p* = 0.001; and GA < 32: 4.3% vs. 0.5%, *p* = 0.001, respectively. For twins, GA < 37: 64% vs. 52%, *p* = 0.001; GA < 34: 27% vs. 15.5%, *p* = 0.001; and GA < 32: 11.2% vs. 6.7%, *p* = 0.006, respectively). Comparing the rates of PTB < 37 weeks, PTB < 34 weeks and PTB < 32 weeks between the OD and IVF groups for singletons, a trend was seen for a higher rate of PTB < 37 weeks among the OD group (24% vs. 20.8%, *p* = 0.08). Higher rates of PTB < 34 weeks and PTB < 32 weeks were found among the OD group (GA < 34: 7.5% vs. 5.3%, *p* = 0.04 and GA < 32: 4.3% vs. 2.7%, *p* = 0.02, respectively). A trend was demonstrated for a higher rate of PTB < 37 weeks among the OD vs. the IVF group (64% vs. 58%, *p* = 0.06). The rate of PTB < 34 weeks was higher among the OD vs. the IVF group (27% vs. 18.5%, *p* = 0.002). Regarding triplets, the rates of PTB < 32 weeks were lower in the OD group compared to the IVF and SP groups. The OD group had lower birthweights and higher rates of SGA compared to the SP group for singletons (2923.2 ± 674 g vs. 3262.3 ± 495 g, *p* = 0.001 and 9.7% vs. 4.6% *p* = 0.001, respectively). Similar birthweights and SGA rates were found among the OD and IVF groups for singletons, twins and triplets (Table 2).

### 3.3. Obstetrical Outcomes

The obstetrical outcomes are depicted in Table 3.

Hypertensive disorders of pregnancy were significantly higher among the OD group compared to the IVF and SP groups for singletons (4.3% vs. 1.7% and 0.7%) and for twins (7.5% vs. 4.3% and 3.4%).

Among singletons, a higher rate of DM/GDM was found among OD pregnancies vs. SPs (2.1% vs. 0.5%, *p* = 0.001), while the rate in the OD and IVF groups was similar. Among twin pregnancies, the OD group had a higher rate of diabetes vs. the IVF and SP groups (6.7% vs. 3.9% and 2.8%). Malpresentation occurred more often in OD vs. SP singletons (1.9% vs. 0.5%, *p* = 0.001). The rates of malpresentation were similar between OD and IVF pregnancies for singletons and twins.

Multivariable logistic regressions for SGA and PTB < 37 weeks are demonstrated in Table 4.

The multivariable logistic regressions for PTB < 37 weeks and SGA among singletons and twins demonstrated that IVF, OD, PIH and diabetes in pregnancy were significant factors (Table 4). The regressions for SGA among singletons demonstrated that IVF, OD and PIH were significant factors, and for SGA among twins, PIH was a significant factor.

## 4. Discussion

This study reports the increased risk of several obstetric and perinatal outcomes in OD pregnancies when compared to IVF and spontaneous singleton, twin and triplet pregnancies. Our findings indicated higher prevalence of PTB, hypertensive diseases of pregnancy and diabetes among pregnancies resulting from OD.

The OD group was characterized by significantly older maternal age. Although initially used to treat patients with premature ovarian insufficiency (POI), advanced maternal age has become a leading indication for OD due to depleted ovarian reserves [22]. This finding is also supported by the American Society of Reproductive Medicine [2].

Among singletons and twins, we found OD to be associated with the highest risk of PTB compared to IVF and SP, with a significantly lower mean GA compared to SP. Furthermore, the risk for PTB before 37, 34 and 32 weeks of gestation for both singletons and twins was consistently higher in the OD group. Data regarding the risk for PTB associated with OD pregnancies are inconsistent. While Rodriguez-walleberg et al. and Guilbaud et al. failed to demonstrate an increased risk for PTB among OD pregnancies vs. autologous IVF pregnancies and SP in singletons and twins [12,17], Storgaard et al. and Kamath et al. demonstrated an increased risk of PTB among OD pregnancies [9,11]. Our findings regarding PTB are consistent with those of Storgaard et al., Kamath et al. and others, demonstrating a high rate of PTB among OD pregnancies both in singleton and twin gestations [7,9,11,18,19].

Multivariable logistic regression analysis to evaluate confounders also strengthened these results and demonstrated that OD and IVF were independent, significant factors related to PTB < 37 weeks among singleton and twin pregnancies.

Among singletons, higher rates of SGA were found among the OD and IVF groups compared to the SP group. Despite moderate-quality evidence that the risk of SGA in singletons is not increased when comparing OD pregnancies to SPs [9], the high risk of SGA associated with ART pregnancies has been previously reported [23,24]. The poorer outcomes following ART treatments are multifactorial and may be related to the IVF technique, to the multiple corpora lutea which may result in abnormal placentation, and perhaps to the infertility disorder itself [24]. A multivariable logistic regression analysis for SGA in singletons showed that both OD and IVF were significant variables.

Upon examining maternal complications in singleton pregnancies, OD was associated with an increased risk for PIH compared to IVF and SP both in singletons and twins. PIH is well known to be associated with perinatal complications, such as PTB and SGA. Regardless of the context, these complications are well known to occur in hypertensive disorders of pregnancy [25]. The high rate of PIH among the OD group reinforces previous studies demonstrating similar results [6,7,9,10,16,26]. Klatsky et al. demonstrated that pregnancies derived from OD are at higher risk for hypertensive disorders of pregnancy, with 24.7% among OD recipients vs. 7.4% among women undergoing IVF with autologous oocytes [6]. In a meta-analysis, Masoudian et al. showed an increased risk of pre-eclampsia and gestational hypertension among OD pregnancies compared with other ART methods or natural conception [26]. Rizzello et al. also demonstrated an increased risk of PIH among OD vs. IVF pregnancies (aOR 2.7) and OD pregnancies vs. SPs (aOR 3.6) [10]. One theory proposed to explain the higher rate of PIH among oocyte recipients is an abnormal immunological response to the allogenic fetal antigens, which may play a role in the pathogenetic mechanism of hypertensive placental disorders in OD pregnancies. The immunological differences in the fetus could be the cause, leading to an allograft reaction [26,27]. Some studies suggest that HLA incompatibility may play an important role, considering OD to be a form of “transplantation” without immunosuppression [28]. Furthermore, lesions of chronic inflammation were found more often in the placentae of OD pregnancies compared to those of spontaneous or IVF pregnancies. This hypothesis of chronic placental inflammatory lesions can also support maternal anti-fetal rejection [29].

Finally, maternal age, which was significantly higher in the OD group, may itself result in increased risk for PIH, as well as diabetes.

A higher rate of diabetes among OD singleton and twin pregnancies was previously described in the literature. Sheffer-Mimouni et al. reported a 24% rate of GDM among OD pregnancies [25]. In a review and meta-analysis, Moreno-Sepulveda et al. showed that OD is associated with a higher risk of GDM, PIH, PTB, low birth weight and Cesarean section [30].

Higher rates of malpresentation were demonstrated among OD and IVF pregnancies compared to SP. In general, higher rates of malpresentation are known to occur among IVF pregnancies. This may be due to anatomical factors related to infertility, along with other perinatal complications [31,32].

Regarding the analysis of triplets, except for maternal age and SES, which were significantly higher among the OD group, and mean GA, which was also higher among the OD compared to IVF pregnancies, all other comparisons for triplets, including perinatal and obstetric outcomes, were not significant. Data regarding obstetrical outcomes among triplets are not presented due to the small number of events.

### Strengths and Limitations

To the best of our knowledge, this study includes one of the largest sample sizes that has been investigated to address the perinatal outcomes of OD pregnancies and is the first to provide information regarding triplets.

However, this study also had some limitations. Due to the retrospective design, the data were based on diagnoses assigned by different physicians; thus, some information was under-reported, especially obstetrical outcomes based on ICD-9 diagnoses, which explains the lower rates of obstetrical complications. Furthermore, our database did not include information regarding indications for OD. To clarify this topic, further study is required to assess the relation between the indications for OD pregnancies and obstetrical outcomes. Considering the validity and applicability of our results, the study population represents a highly heterogenic and versatile population in our country with regard to age, race and ethnicity. Thus, we assume that our results could be cautiously applied to other populations, although further studies are necessary to validate our results. Finally, the data regarding triplets should be interpreted with caution because the SP group may include cycles of ovulation induction as well as spontaneous conceptions.

## 5. Conclusions

Pregnancies following OD should be considered high-risk, primarily due to the more advanced maternal age of the women, with increased risks of PIH, PTB and diabetes in both singleton and twin gestations, and more SGA neonates among singletons. Careful follow-ups of women with OD pregnancies should be carried out, including cervical length surveillance to identify risk for preterm labor, frequent blood pressure measurements to identify PIH, more frequent glucose surveillance, and perhaps the administration of low-dose aspirin to reduce the risk of PE.

## Figures and Tables

**Table 1:** Maternal and clinical characteristics of women after oocyte donation, IVF and spontaneous pregnancies.

Characteristic	OD	IVF	SP	*p* Value OD vs. IVF	*p* Value OD vs. SP	*p* Value IVF vs. SP
Singleton						
Number of neonates	483	12,579	456,072			
Maternal age, years, mean (SD)	39.7 (4.1)	34.5 (4.8)	31.7 (5.3)	0.001	0.001	0.001
BMI, mean (SD)	25.5 (5.2)	25.8 (5.8)	25.2 (5.2)	0.235	0.258	0.001
SES, mean (SD)	7 (1.5)	6.7 (2)	5.9 (2)	0.001	0.001	0.001
Nulliparous, n (%)	314 (65)	75 (0.6)	236,245 (51.8)	0.001	0.032	0.001
Twin						
Number of neonates	240	5411	9334			
Maternal age, years, mean (SD)	39 (4.6)	32.6 (4.4)	31.2 (5.1)	0.001	0.001	0.001
BMI, mean (SD)	26 (5.7)	26 (6.1)	25.5 (5.6)	0.984	0.122	0.001
SES, mean (SD)	7.2 (1.6)	6.6 (1.8)	6.4 (2)	0.001	0.001	0.001
Nulliparous, n (%)	NA	NA	NA	NA	NA	NA
Triplet						
Number of neonates	15	159	252			
Maternal age, years, mean (SD)	35.6 (2.5)	32 (3.9)	29.7 (5)	0.001	0.001	0.001
BMI, mean (SD)	29 (6.8)	27 (6.5)	26.8 (6.6)	0.248	0.2	0.810
SES, mean (SD)	7.2 (0.4)	5.8 (1.8)	5 (1.8)	0.006	0.001	0.001

SES—socioeconomic status.

**Table 2:** Perinatal outcomes of children born after oocyte donation, IVF and spontaneous pregnancy.

Characteristic	OD	IVF	SP	*p* Value OD vs. IVF	*p* Value OD vs. SP	*p* Value IVF vs. SP
Singleton						
Number of neonates	483	12,579	456,072			
GA, weeks, mean (SD)	37.5 (3)	37.6 (2)	39 (2)	0.155	0.001	0.001
GA < 37, n (%)	116 (24)	2616 (20.8)	25,540 (5.6)	0.089	0.001	0.001
GA < 34, n (%)	36 (7.5)	666 (5.3)	456 (1.1)	0.042	0.001	0.001
GA < 32, n (%)	21 (4.3)	339 (2.7)	2280 (0.5)	0.027	0.001	0.001
Birth weight, g, mean (SD)	2923.2 (674)	2916 (611)	3262.3 (495)	0.818	0.001	0.001
SGA, n (%)	46 (9.7)	1195 (9.5)	20,979 (4.6)	0.842	0.001	0.001
Female (%)	236 (49)	6364 (50.6)	234,877 (51.5)	0.518	0.288	0.043
Twin						
Number of neonates	240	5411	9334			
GA, weeks, mean (SD)	35 (3)	35.4 (3)	36 (2.5)	0.004	0.001	0.001
GA < 37, n (%)	153 (64)	3138 (58)	4853 (52)	0.065	0.001	0.001
GA < 34, n (%)	64 (27)	1001 (18.5)	1446 (15.5)	0.002	0.001	0.001
GA < 32, n (%)	27 (11.2)	454 (8.4)	625 (6.7)	0.115	0.006	0.001
Birth weight, grams, mean (SD)	2205 (491)	2252 (524)	2332 (527)	0.172	0.001	0.001
SGA, n (%)	9 (3.8)	367 (6.8)	551 (5.9)	0.063	0.155	0.033
Female, n (%)	129 (54)	2710 (50.1)	4788 (51.3)	0.216	0.387	0.141
Triplet						
Number of neonates	15	159	252			
GA, weeks, mean (SD)	34 (1)	32.2 (3)	33 (2.4)	0.013	0.235	0.001
GA < 37, n (%)	15 (100)	159 (100)	237 (94)	NA	0.333	0.002
GA < 34, n (%)	7(47)	90 (56)	114 (45)	0.508	0.891	0.033
GA < 32, n (%)	(0)	48 (30)	66 (26)	0.014	0.022	0.481
Birth weight, g, mean (SD)	1920 (409)	1673 (460)	1772 (432)	0.023	0.196	0.003
SGA, n (%)	2 (12)	18 (11.2)	28 (11)	0.810	0.869	0.84
Female, n (%)	9 (54)	84 (52)	111 (44)	0.915	0.484	0.121

GA, gestational age; SGA, small for gestational age: below tenth percentile.

**Table 3:** Maternal complications of women after oocyte donation, IVF and spontaneous pregnancies.

Characteristic	OD	IVF	SP	*p* Value OD vs. IVF	*p* Value OD vs. SP	*p* Value IVF vs. SP
Singleton						
Number of neonates	483	12,579	456,072			
Hypertensive disorders (HTN, pre-eclampsia, eclampsia, GHTN), n (%)	21 (4.3)	214 (1.7)	3192 (0.7)	0.001	0.001	0.001
DM/GDM, n (%)	10 (2.1)	189 (1.5)	4560 (0.5)	0.360	0.001	0.001
Malpresentation	9 (1.9)	214 (1.7)	4560 (0.5)	0.745	0.001	0.001
Twin						
Number of neonates	240	5411	9334			
Hypertensive disorders (HTN, pre-eclampsia, eclampsia, GHTN), n (%)	18 (7.5)	232 (4.3)	317 (3.4)	0.018	0.001	0.005
DM/GDM, n (%)	16 (6.7)	211 (3.9)	261 (2.8)	0.031	0.001	0.001
Malpresentation, (n (%)	16 (6.7)	389 (7.2)	485 (5.2)	0.735	0.309	0.001
Triplet						
Number of neonates	15	159	252			
Hypertensive disorders (HTN, pre- eclampsia, eclampsia, GHTN), n (%)	0 (0)	12 (7.5)	24 (9.5)	0.274	0.212	0.48
DM/GDM n (%)	0 (0)	3 (2)	12(4.8)	0.595	0.389	0.128
Malpresentation	0 (0)	21 (13)	30 (12.3)	0.136	0.15	0.807

HTN, hypertension; GHTN, gestational hypertension; DM, diabetes mellitus; GDM, gestational diabetes mellitus.

**Table 4:** Multivariable logistic regression for SGA < 10% and PTB < 37 weeks.

	SGA < 10%				PTB < 37 weeks			
Variable	OR	95% CI		*p*-Value		95% CI		*p*-Value
		Lower	Upper		OR	Lower	Upper	
Singleton								
Maternal age, years	1.002	0.99	1	0.001	1.01	1.012	1.018	0.001
Body mass index, kg/m^2^	0.95	0.94	0.95	0.001	0.99	0.98	0.99	0.001
Socioeconomic status	0.94	0.93	0.95	0.001	1	0.99	1.01	0.301
In vitro fertilization	2.25	2	2.41	0.001	4.35	4.13	4.6	0.001
Oocyte donation	1.9	1.3	2.66	0.001	4.14	3.3	5.2	0.001
Hypertension	3.4	3	3.8	0.001	5	4.56	5.56	0.001
Diabetes (DM, GDM)	1	0.8	1.2	0.978	2.63	2.3	2.9	0.001
Twin								
Maternal age, years	1.02	1	1.04	0.004	0.98	0.97	0.99	0.001
Body mass index, kg/m^2^	0.97	0.96	0.99	0.005	0.99	0.98	0.99	0.016
Socioeconomic status	0.98	0.96	0.99	0.811	1.03	1.02	1.06	0.001
In vitro fertilization	1.13	0.95	1.3	0.158	1.3	1.19	1.42	0.001
Oocyte donation	0.44	0.19	1.04	0.061	1.38	1	1.9	0.05
Hypertension	1.47	1	2.15	0.046	2.4	1.9	3.1	0.001
Diabetes (DM, GDM)	1.25	0.84	1.88	0.268	1.7	1.36	2.2	0.001

OR, odds ratio; CI, confidence interval; DM diabetes mellitus, GDM, gestational diabetes mellitus.

## Data Availability

The data presented in this study are available on request from the corresponding author due to the privacy of data.
